# Supplementary material for: Dietary Supplementation with a Blend of Hydrolyzable and Condensed Tannins Ameliorates Diet-Induced Intestinal Inflammation in Zebrafish (Danio rerio)
Source: Animals (Basel). 2022 Dec 31;13(1):167. doi: 10.3390/ani13010167 (PMC9818001; doi:10.3390/ani13010167)
Supplement: Supplementary file 1 [file animals-13-00167-s001.zip › animals-2078173-supplementary.pdf]

Table S1. Primer sequences for qRT-PCR.

Gene	Sequence 5′-3′	Reference
beta actin	F: GCAGAAGGAGATCACATCCCTGGC	https://doi.org/10.3390/nu13030998
	R: CATTGCCGTCACCTTCACCGTTC	
cox2	F: CCCTGTCAGAATCGAGGTGT	https://doi.org/10.1038/nchembio.147
	R: TTGGGAGAAGGCTTCAGAGA	
il1b	F: GGACTTCGCAGCACAAAATGAA	https://doi.org/10.1101/2020.04.09.033837
	R: TTCACTTCACGCTCTTGGATGA	
cxcl8-l1	F: GTCGCTGCATTGAAACAGAA	https://doi.org/10.4049/jimmunol.1203266
	R: CTTAACCCATGGAGCAGAGG	
tnfα	F: GGGCAATCAACAAGATGGAAG	https://doi.org/10.1038/s41598-018-28511-w
	R: GCAGCTGATGTGCAAAGACAC	

Table S2. BW increment (mg), VFI (mg), and FCR observed during the experimental period for the considered treatments.

Parameters	BW increment		VFI		FCR	
**Treatments**	**m**	**ds**	**m**	**ds**	**m**	**ds**
Control	59,1	3,3	13,2	0,9	2,7	0,3
TSP 12	58,2	3,3	14,5	0,9	2,8	0,3
TSP 24	65,1	3,0	13,1	0,8	2,4	0,1
TSP 48	64,3	2,8	14,5	0,9	2,7	0,2
SEM	0.894		0,261		0,063	
P	0,0578		0,1687		0,0733	

*SEM: Standard error of the mean.

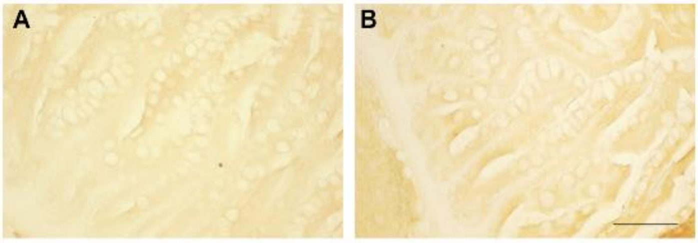

Figure S1. Antibodies specificity. Negative controls performed by omitting the primary antibodies for TNFα (**A**) or COX2 (**B**). Scale bar: 100 μm.

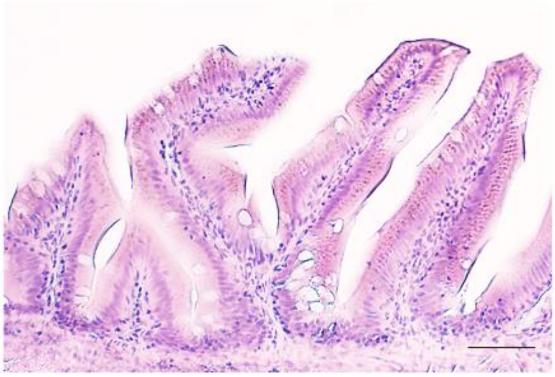

Total morphological alterations score (n)	4 ± 1
Mean number of Goblet cells/villus (n)	7 ± 2

Figure S2. Hematoxylin and eosin (H&E) staining of intestine of zebrafish fed a diet based on Artemia salina cysts. Scale bar: 100 μm. In the table, the score number of the morphological alteration and the number of goblet cells/villus in the same zebrafish are reported.
